# Correction: Seasonality of ventricular fibrillation at first myocardial infarction and association with viral exposure

**DOI:** 10.1371/journal.pone.0232569

**Published:** 2020-04-27

**Authors:** Charlotte Glinge, Thomas Engstrøm, Sofie E. Midgley, Michael W. T. Tanck, Jeppe Ekstrand Halkjær Madsen, Frants Pedersen, Mia Ravn Jacobsen, Elisabeth M. Lodder, Nour R. Al-Hussainy, Niels Kjær Stampe, Ramona Trebbien, Lars Køber, Thomas Gerds, Christian Torp-Pedersen, Thea Kølsen Fischer, Connie R. Bezzina, Jacob Tfelt-Hansen, Reza Jabbari

The fifteenth author's initials are indexed incorrectly in PubMed. The correct initials are: Fischer TK.
